# Supplementary figures and images for: Microglial SIRPα regulates the emergence of CD11c+ microglia and demyelination damage in white matter
Source: eLife. 2019 Mar 26;8:e42025. doi: 10.7554/eLife.42025 (PMC6435324; doi:10.7554/eLife.42025)

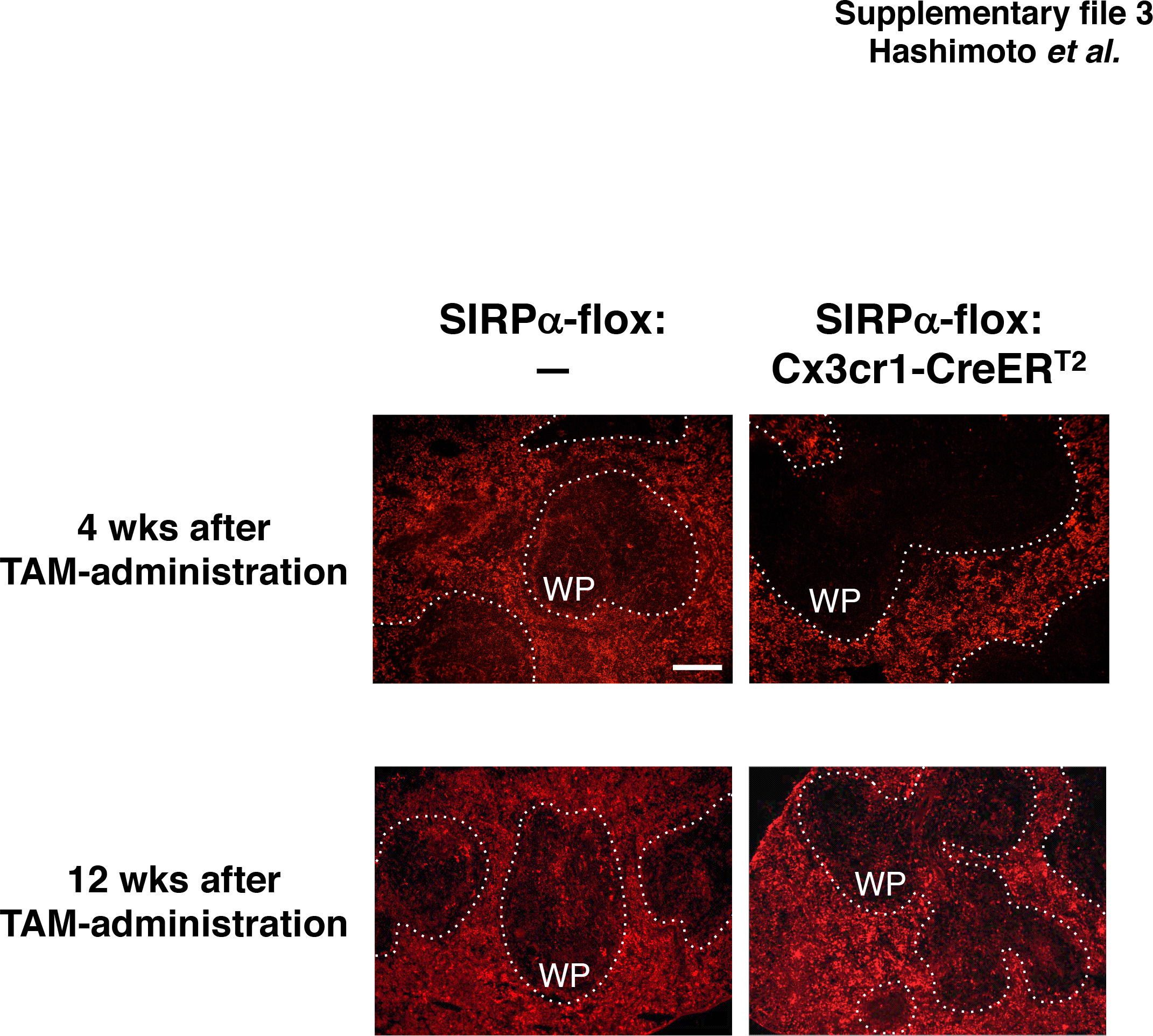

Supplement: Supplementary file 3. — Spleens were isolated from control (SIRPα-flox:—) and SIRPα cKO (SIRPα-flox:Cx3cr1-CreERT2) mice 4 (upper panels) or 12 (lower panels) weeks after the administration of tamoxifen (TAM). Immunofluorescence staining with specific antibodies to SIRPα (red) are shown. The white pulp areas (WP) were surrounded by a white dotted line. Scale bar: 200 μm. [file elife-42025-supp3.jpg]

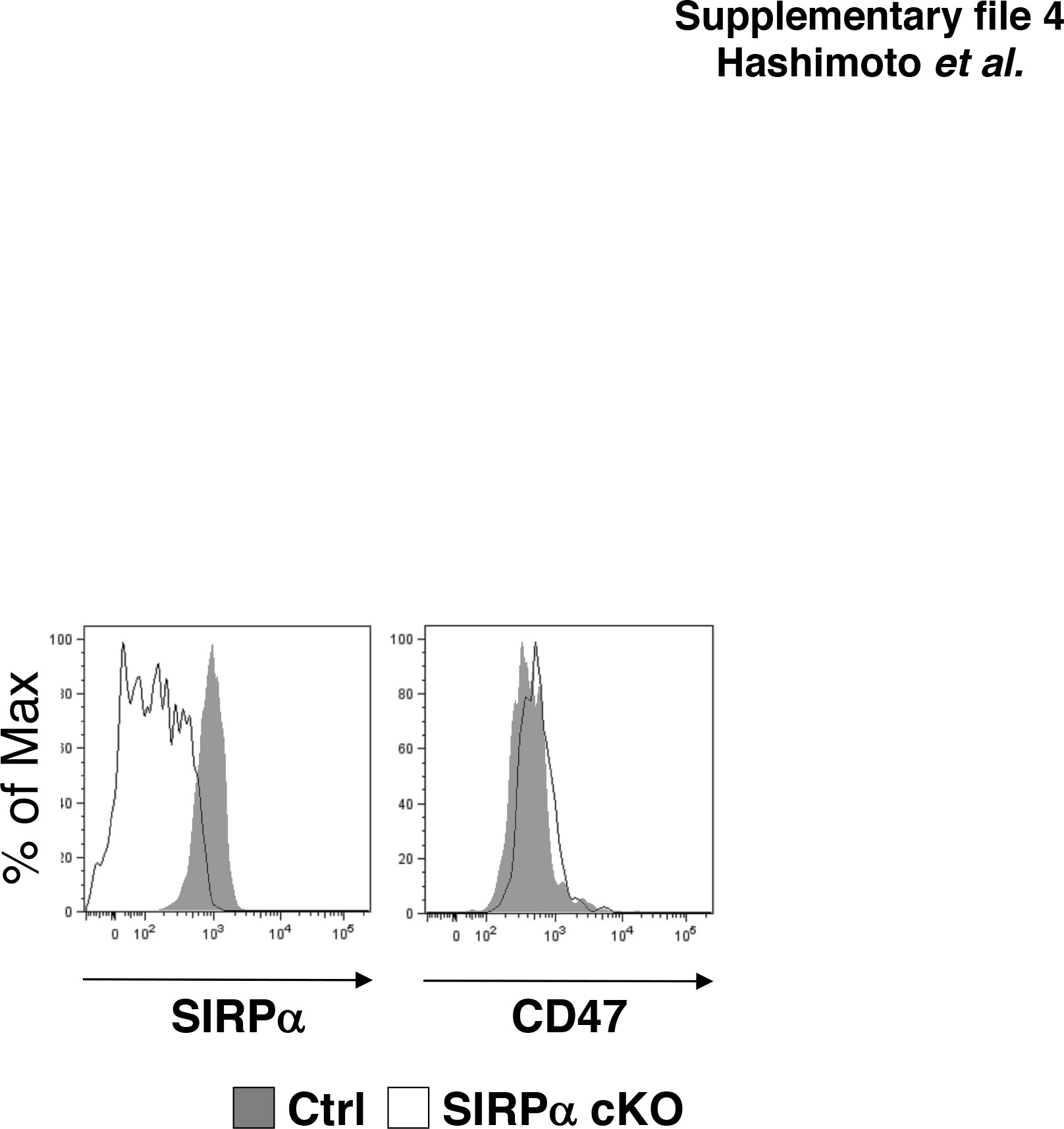

Supplement: Supplementary file 4. — Cells were isolated from the spinal cord of control (SIRPα-flox:— (Ctrl)) or SIRPα cKO (SIRPα-flox:Cx3cr1-CreERT2) mice at 25–28 wks of age, and the expression of SIRPα and CD47 on CD11b+/CD45dim/lo microglia were analysed by flow cytometry. Expression profiles for SIRPα and CD47 in CD11b+/CD45dim/lo microglia are shown. Filled and open traces indicate control and SIRPα cKO mice, respectively. [file elife-42025-supp4.jpg]

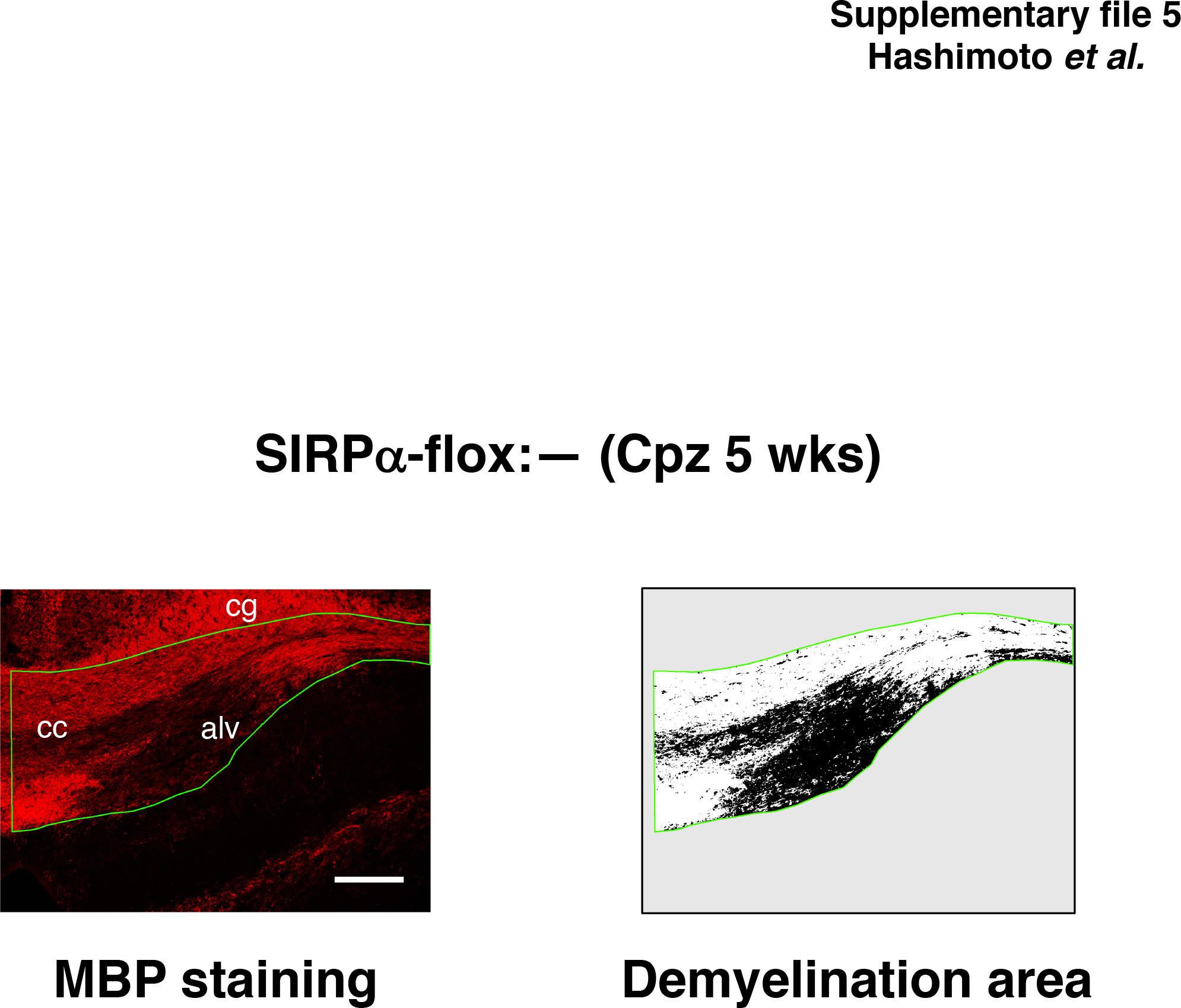

Supplement: Supplementary file 5. — A brain section prepared fromSIRPα-flox:— mice fed with a 0.2% (w/w) cuprizone diet for five weeks (Cpz 5 wks) were subjected to immunofluorescence staining with specific antibodies to MBP (red in the left panel). The white matter area analysed in the image is surrounded by a yellow line. Demyelination area with low MBP-immunoreactivity in the white matter area was shown in the right panel as filled (black) area. Ratio of area size (pixel number) of the demyelination area (black in the right panel) to that of the white matter area (black + white in the right panel) was calculated. alv, hippocampal alveus; cc, corpus callosum; cg, cingulum. Scale bar: 200 μm. [file elife-42025-supp5.jpg]
