# Supplementary material for: Microglial SIRPα regulates the emergence of CD11c+ microglia and demyelination damage in white matter
Source: eLife. 2019 Mar 26;8:e42025. doi: 10.7554/eLife.42025 (PMC6435324; doi:10.7554/eLife.42025)

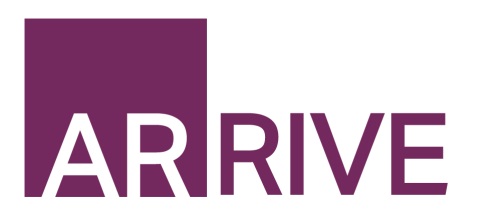


The ARRIVE Guidelines Checklist

Animal Research: Reporting In Vivo Experiments

Carol Kilkenny^1^, William J Browne^2^, Innes C Cuthill^3^, Michael Emerson^4^ and Douglas G Altman^5^

*^1^The National Centre for the Replacement, Refinement and Reduction of Animals in Research, London, UK, ^2^School of Veterinary Science, University of Bristol, Bristol, UK, ^3^School of Biological Sciences, University of Bristol, Bristol, UK, ^4^National Heart and Lung Institute, Imperial College London, UK, ^5^Centre for Statistics in Medicine, University of Oxford, Oxford, UK.*

|  | | ITEM | RECOMMENDATION | Section/ Paragraph |
| --- | --- | --- | --- | --- |
| 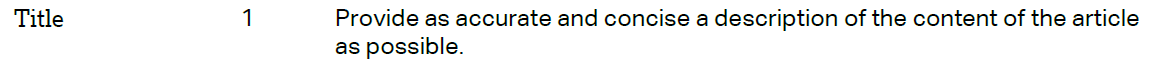 | | | Title |  |
| 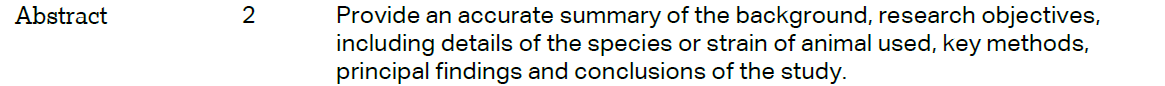 | | | Abstract |  |
| INTRODUCTION | | |  |  |
| 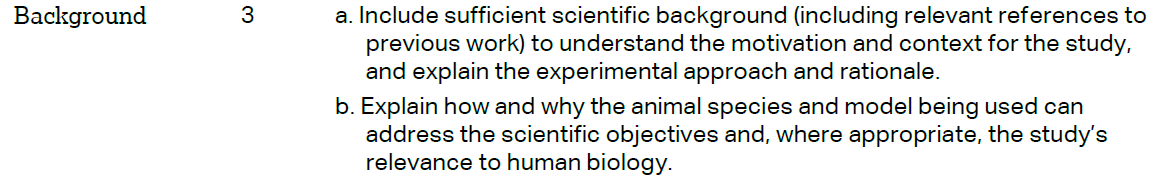 | | | Paragraphs 1 |  |
| 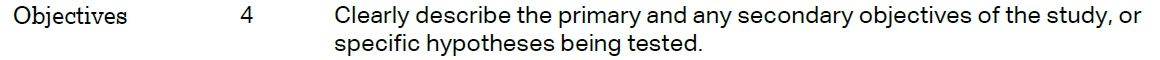 | | | Paragraphs 2 |  |
| METHODS | | |  |  |
| 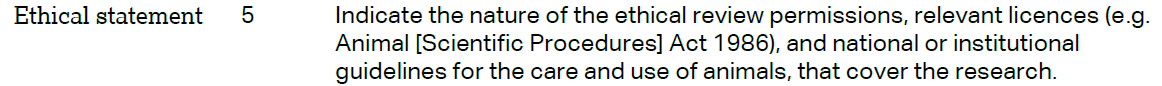 | | | Line 528th-530th  Institutional Approval no. 13-009 |  |
| 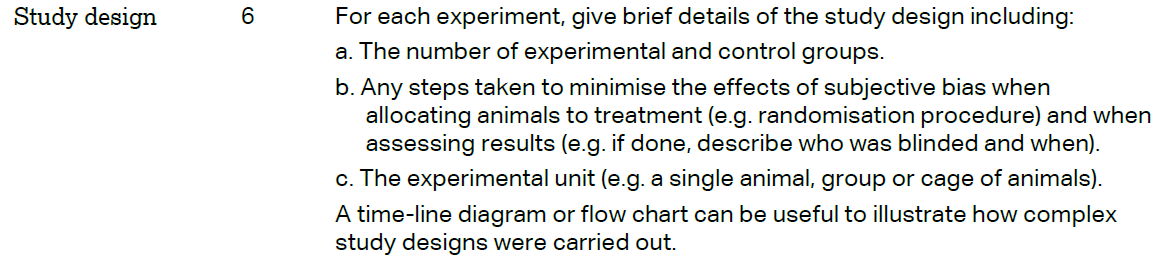 | | | a. Figure legends |  |
| 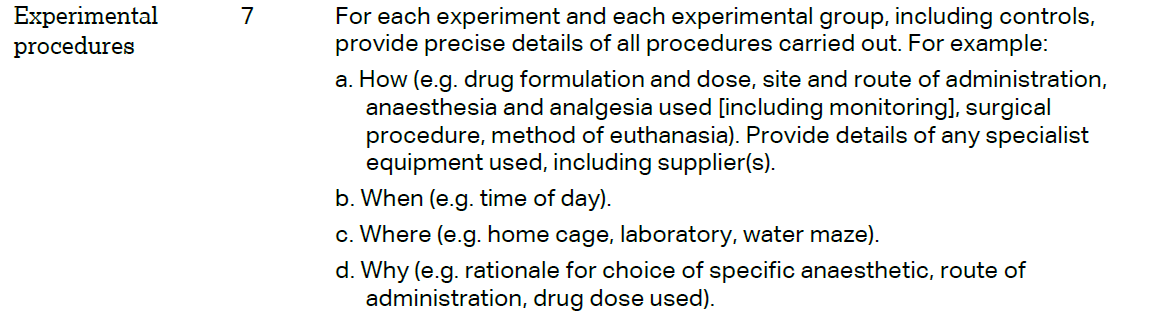 | | | Materials and methods |  |
| 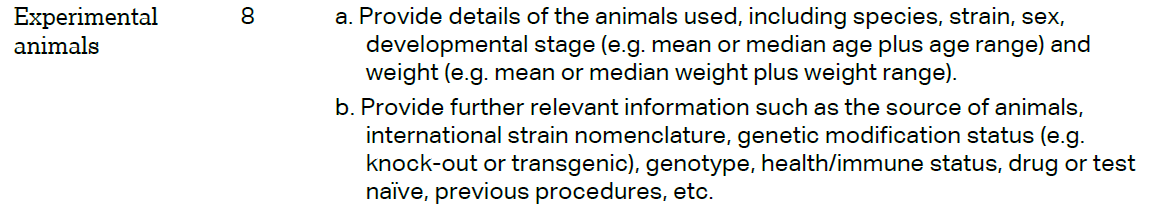 | | | b. Key resources tables in Materials and methods |  |

The ARRIVE guidelines. Originally published in *PLoS Biology*, June 2010^1^

| 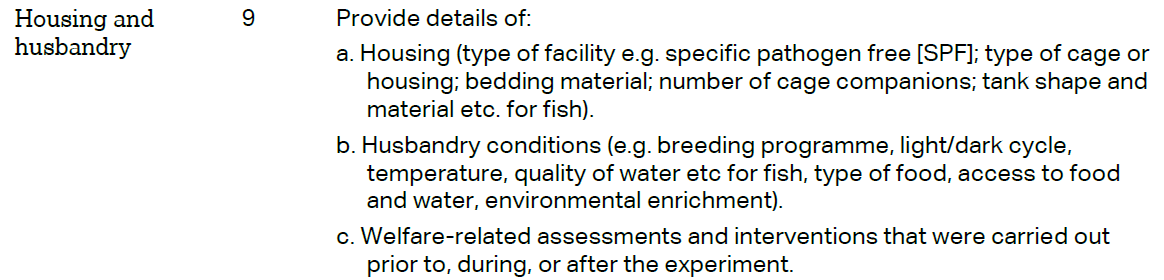 | a. b. Animals in Materials and methods | |
| --- | --- | --- |
| 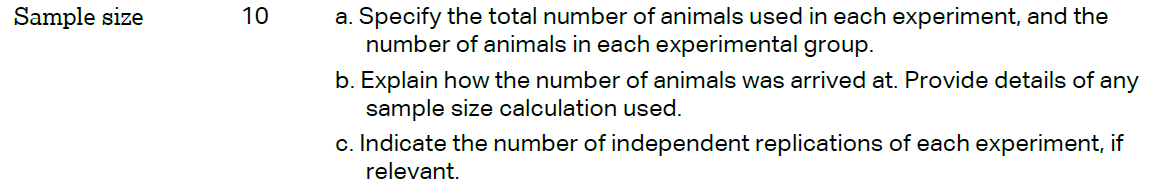 | a. c. Figure legends | |
| 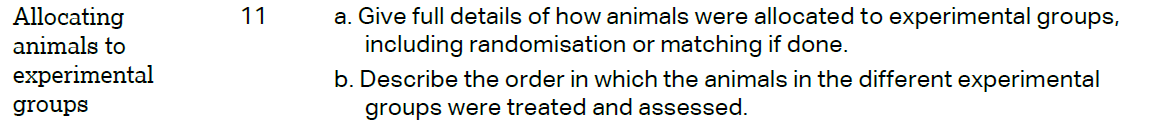 | a. b. Not applicable | |
| 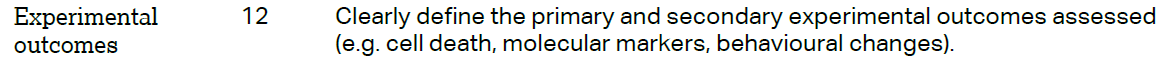 | Materials and methods | |
| 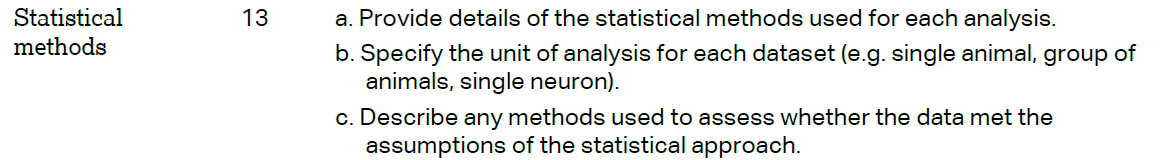 | Statistical analysis in Materials and methods | |
| RESULTS |  | |
| 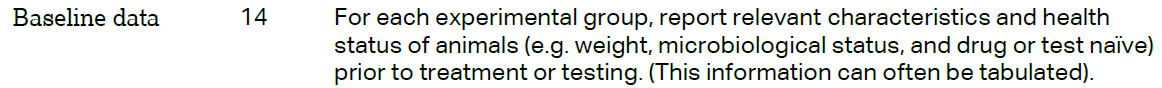 | In Figures | |
| 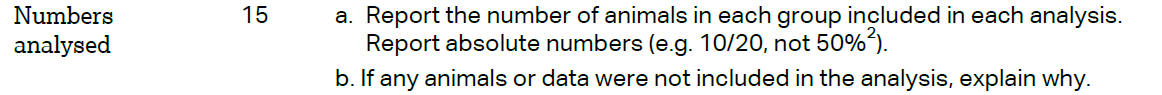 | Figure legends | |
| 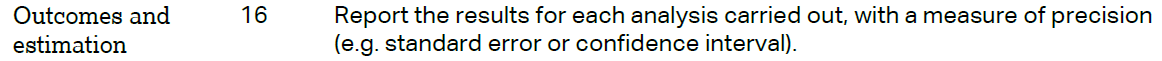 | In Figures  Results section | |
| 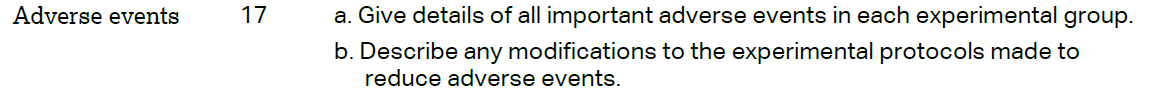 | Histological analysis section in Materials and methods | |
| DISCUSSION |  | |
| 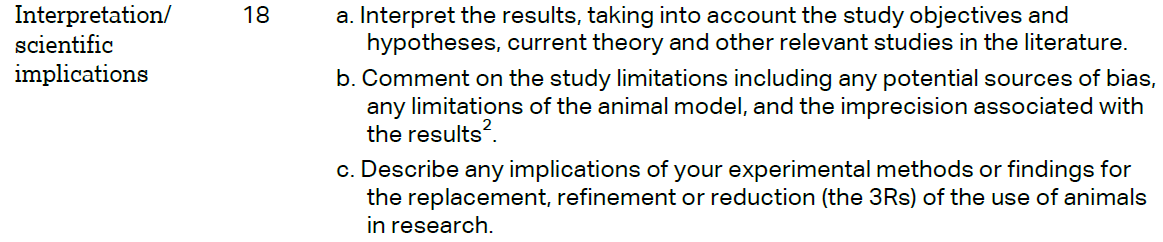 | Discussion | |
| 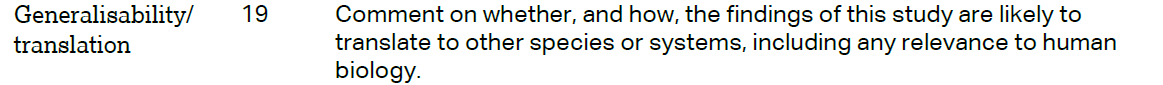 | Discussion | |
| 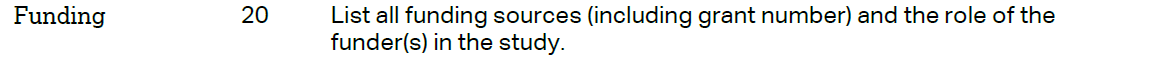 | | Related information were uploaded on website |


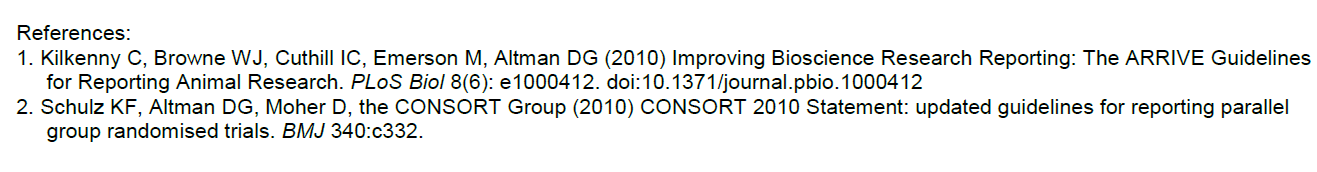

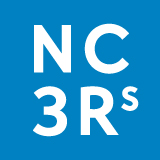

Supplement: Reporting standard 1. [file elife-42025-fig7.docx]
